# Migraine and behavior in children: influence of maternal headache frequency

**DOI:** 10.1007/s10194-012-0441-x

**Published:** 2012-03-30

**Authors:** Marco A. Arruda, Marcelo E. Bigal

**Affiliations:** 1 Glia Institute, Av. Braz Olaia Acosta, 727, s. 310, CEP14026040, Ribeirão Preto, SP Brazil; 2 Merck Investigator Study Program and Scientific Education, Global Center for Scientific Affairs, Office of the Chief Medical Officer, Merck Inc, West Point, PA USA; 3 Department of Neurology, Albert Einstein College of Medicine, Bronx, NY USA

**Keywords:** Headache, Migraine, Psychiatric comorbidity, Maternal headache, Childhood, Epidemiology

## Abstract

We took advantage of a large population study in order to measure child behavior, as captured by the Child Behavior Checklist (CBCL) as a function of headache status in the children and their mothers. Of the target sample, consents and analyzable data were obtained from 1,856 families (85.4 %). Headache diagnoses were defined according to the second edition of the International Classification of Headache Disorders, and behavioral and emotional symptoms were assessed by the validated Brazilian version of the CBCL. We calculated the relative risk of abnormalities in the CBCL domains as a function of headache status in the children, after adjusting by a series of main effect models. Children with migraine were more likely to present abnormal scores in several of the CBCL scales, relative to children without migraine, and maternal migraine status contributed little to the model. However, when the mother had daily headaches, both children with and without migraine had similar CBCL scores. In multivariate analyses, migraine status in the children predicted CBCL scores (*p* < 0.01). Headache status and headache frequency in the mother did not predict CBCL scores in children with migraine but predicted in children without migraine (*p* < 0.01). The burden of migraine to the family is complex. Children with migraine are more likely to have behavioral and emotional symptoms than children without migraine. Children without migraine may be affected, in turn, by frequent headaches experienced by their mothers.

## Introduction

For several neurological disorders, early onset cases often have the highest level of biological risk and a more refractory outcomes [1]. High frequency headaches [2] and migraine [3] are common in pre-adolescent children, offering a strong opportunity for the development of studies assessing determinants of disease onset and progression.

About half of the variance in migraine prevalence is accounted for by genetic factors, leaving a strong role for environmental or non-genetic familial risk factors [4].

Epidemiological studies show that migraine aggregates within families [5–7]. The relative risk of migraine in family members of episodic migraine probands, compared with those of controls, ranges from 1.5 to 19.3 in different casuistics [8–13]. Aggregation seems to increase as a function of disease severity [11], and early onset of migraine in the proband as well as the severity of migraines is associated with higher levels of family aggregation [14]. A recent study has brought some evidence that also the headache frequency aggregates in the family [15]. Frequency of headaches in the mother predicted frequency of headaches in the children; when mother had low frequency headaches, children had increased chance to have low or intermediate headache frequency (relative risk = 1.4, 1.2–1.6) but not very frequent headaches. When mothers had headaches on more days than not (chronic daily headaches), risk of frequent headaches in the child were increased by almost 13-fold. These findings are intriguing and may non-exclusively suggest that biological predisposition drives migraine frequency, or that shared environment exposures influence headache frequency in individuals sharing the household.

Comorbidities seem to play a role in migraine onset, progression and response to therapies, and several behavioral and emotional problems have been reported in children with migraine. Clinical [16–19] and populational studies [20, 21] suggest that, relative to children without headaches, those with migraine are more likely to have somatic, anxiety, and depressive symptoms. Limited findings also suggest that pediatric migraine is associated with impaired attention span [21] and hyperactivity–impulsivity [22], but not with fully developed attention deficit hyperactivity disorder (ADHD) [22].

Nonetheless, studies accounting for the influence of maternal headache status and of headache frequency on the comorbid association are not available. Accordingly, herein, we take advantage of a large population study to measure child behavior, as captured by the Child Behavior Checklist (CBCL), as a function of headache status in the children and their mothers.

## Methods

### Overview

This study was conducted as part of a large ongoing population study aiming to investigate mental health and headache in children and adolescents (Attention-Brazil Project) and details of the project have been described elsewhere [23]. In brief, the project consists of two phases. In Phase 1, we piloted the methods by targeting all children from 5 to 12 years registered in the public school system of a city (Santa Cruz das Palmeiras, São Paulo, Brazil). Phase 2 (currently ongoing) draws national representation to the sample. The data reported here is not being investigated in Phase 2.

### Geographic characterization and target sample

According to the demographic census, the studied region covers an area with 32,862 inhabitants (year of 2008). Of them, 30,387 (92.4 %) are in the urban area. Although the demographic census does not distinguish the age range from 5 to 12 years, there are 5,055 children from 5 to 14 years (15.3 % of the population). Life expectancy is 73.71 years, and fecundity rate is 2.13, rates that are similar to the Brazilian rates [24].

A total of 2,173 children were younger than 12 years and were registered in the elementary school; children registered in the middle school were not included in this study. Therefore, although all children younger than 11 years were targeted, only a subsample of those aged 11 and 12 were included.

Direct interviews were conducted for the mother or caregiver and for the teachers. Both children from urban and rural areas were assessed, as long as they were enrolled in the school system (which is mandatory). Of 2007 potential participants, consent was obtained from 1,994 (91 %) and analyzable data (complete demographics, mental health and headache information) were obtained from 1,856 children (85.4 %). The very high participation rate is explained by the active engagement of the city authorities in raising awareness about the study (see below).

### Flow of the study

In February of 2009, during the planning for the 2009 school year, all teachers of the public school system were trained by one of us (MAA). They were given information about the study and educated about how to teach the parents about fulfilling the questionnaire (see below). Parents were then invited to attend a meeting at school (during the first week of the school year) and, under the supervision of the teachers which, in turn, were supervised by one of the authors of this study (MAA), fulfilled the questionnaire. Meanwhile, children remained with monitors, practicing physical activities.

### Questionnaires

Parents or guardians (usually the mother) were requested to fulfill the questionnaires on demographics, mental health and headache information about the children, as well as headache information about themselves.

### Headache diagnosis

The headache module of the questionnaire consisted of 14 questions, assessing the distinguishing features required for headache diagnosis of the children and their mothers according to the classification criteria of the Second Edition of the International Classification of Headache Disorders (ICHD-2) [25]. Diagnosis of chronic migraine was defined according to the 2006 appendix of the ICHD-2 [26]. The headache module is the Portuguese version of the questionnaire used in the American Migraine Studies [27] and has been validated [28].

### Behavior and emotional symptoms

Two main approaches to assess behavioral symptoms at childhood exist. Categorical diagnosis that describe psychopathological states as distinct syndromes and dimensional approaches that view psychopathology as a deviance from normal with no clear threshold between subjects with and without a disorder [29]. The latter is the approach of the CBCL adopted by us. An advantage of this approach is to avoid stigmatization and labeling, common risks when conducting behavioral research. The CBCL was developed by Achenbach [30] for the assessment of competencies and psychopathological symptoms in children. The CBCL has been translated into over 60 languages and has been validated in numerous cross-cultural studies [29].

The validated Brazilian version of the CBCL [31] was applied. The competency score of the CBCL consists of 20 questions concerning school achievement, social, and activity scores. From these 3 scales, a total competency score is obtained. The behavior and emotional symptoms score consists of 112 questions focusing on the previous 6 months, which determine 8 symptom scales: ‘Withdrawal’, ‘Somatic complaints’, ‘Anxiousness/Depression’, ‘Social problems’, ‘Thought problems’, ‘Attentional problems’, ‘Delinquent behavior’, and ‘Aggressive behavior’. A ninth scale focusing on ‘sexual problems’ was not included. Two composite scales, Internalizing and Externalizing, were determined. The internalizing scale comprises the ‘Withdrawal’, ‘Somatic complaints’ and ‘Anxiousness/Depression’ scales. The externalizing scale is composed of the ‘Delinquent’ and ‘Aggressive’ behavior scales. The sum of scores of all scales defines the total problem score. The behavior-related factors differ according to age and gender. ‘Clinical relevance’ of behavioral and emotional problems was defined as a CBCL total problem score ≥70 [30].

### Analyses

Sex-specific 1-year prevalence of headache diagnoses was derived by age, race, and income (and used in the analyses, although not presented in full detail to keep the paper focused). To characterize the sample, descriptive statistics were performed. Crude and adjusted prevalence ratios were obtained using binary regression model. Prevalence ratios and 95 % confidence intervals compared specific categories (e.g., age categories or race) with the reference category.

For all contrasts children without headaches of mother without headaches were specified as the reference. We first calculated the relative risk of abnormalities in the CBCL domains as a function of headache status in the children, in crude analyses. We then developed multivariate models estimating CBCL scores as a function of headache status in the mother, children, and of headache frequency in the children after adjusting for demographics, and headache symptoms (nausea, photophobia, phonophobia, severity of pain).

### Investigation review board approval

This study and surveys received full approval from a Human Research Committee (School of Medicine at São José do Rio Preto Medical School, São Paulo, Brazil). Written informed consents were obtained.

## Results

Table 1 displays the demographics of the participating sample and also of those without complete data. Around 52 % of respondents were boys, and most were from the middle class (income class C). Participation rates were very high for all the categories although decreased as a function of decreased family income.

**Table 1:** Demographics of the sample and response rates

	Respondents		Non-respondents		Response rate (%)
	*n*	%	*n*	%	
Age					
5	90	4.8	13	8.6	87.4
6	350	18.9	18	11.9	95.1
7	310	16.7	39	25.8	88.8
8	370	19.9	33	21.9	91.8
9	465	25.1	27	17.9	94.5
10+	271	14.6	21	13.9	92.8
Gender					
Girls	897	48.3	65	43.0	93.2
Boys	959	51.7	86	57.0	91.8
Race					
White	1,082	58.3	64	42.4	94.4
Non-white	699	37.7	62	41.1	91.9
Not stated	75	4.0	25	16.6	75.0
Income class					
A, B	329	17.7	16	10.6	95.4
C	976	52.6	56	37.1	94.6
D, E	551	29.7	79	52.3	87.5
Total	1,856		151		92.5

Of the assessed children, 345 (18.6 %) had not experienced any headache in the past year, and 118 (6.3 %) met full criteria for migraine with and without aura. The overall prevalence of migraine was 6.4 %, being 6.1 % in boys and 6.6 % in girls (non-significant difference). Prevalence was 6.0 % in white children and 7.3 % in non-white (non-significant difference). Prevalence increased with age. Using the age of 5–6 as the reference (3.2 %), prevalence was numerically increased in all subsequent ages, and significantly increased at the age 7–8 (6.2 %, RR = 1.9, 95 % CI = 1.1–3.5) and of 9 or older (8.4 %, RR = 2.6, 95 % CI = 1.5–4.7) (Table 2).

**Table 2:** Prevalence of headache and migraine by age, gender, race and income

	No headache			Migraine with and without aura		
	*n*	%	Relative risk (95 % confidence interval)	*n*	%	Relative risk (95 % confidence interval)
Age						
5, 6	109	24.8	Reference	14	3.2	Reference
7, 8	127	18.7	0.7 (0.6–0.9)	42	6.2	1.9 (1.1–3.5)
9, 10+	109	14.8	0.6 (0.5–0.8)	62	8.4	2.6 (1.5–4.7)
Gender						
Female	159	17.7	Reference	55	6.1	Reference
Male	186	19.4	1.1 (0.9–1.3)	63	6.6	1.1 (0.7–1.5)
Race						
White	201	18.6	Reference	65	6.0	Reference
Non-white	126	18.0	1.0 (0.8–1.2)	51	7.3	1.2 (0.8–1.7)
Not stated	18	24.0	1.3 (0.8–2.0)	2	2.7	0.4 (0.1–1.8)
Income class						
A, B	49	14.9	Reference	13	3.9	Reference
C	178	18.2	1.2 (0.9–1.6)	66	6.8	1.7 (1.0–3.1)
D, E	118	21.4	1.4 (1.1–1.9)	39	7.1	1.8 (1.0–3.3)
Total	345	18.6		118	6.4	

In preliminary analyses, not including maternal headache status, multivariate analyses found a significant relationship between any headache (*p* < 0.05) and migraine headaches (*p* < 0.01) with internalizing problems and with total CBCL score dysfunction (*p* < 0.01). Other variables did not contribute significantly to the model.

Table 3 displays the prevalence of clinical scores in different domains of the CBCL as a function of headache status in the children and their mothers. As contrasted to controls (children without headaches of mother without headaches), children without headache of mother with migraine did not present significant difference in any CBCL domains. Comparing to controls, children with migraine of mother without headaches were more likely to have abnormal scores in the following domains of CBCL (relative risk and confidence intervals are displayed on the table only for ease of reading): somatic (20.3 vs. 3.0 %), anxiety-depressive (12.6 vs. 3.4 %), attention (15.9 vs. 6.1 %), internalizing (49.2 vs. 17.9 %) and total score (32.5 vs. 14.5 %). Relative to controls, children with migraine of mother with migraine had significant differences in the same domains: somatic (23.8 vs. 3.0 %), anxiety-depressive (18.2 vs. 3.4 %), attention (18.8 vs. 6.1 %), internalizing (54.1 vs. 17.9 %) and total score (33.1 vs. 14.5 %), increased by the social domain (12.2 vs. 5.4 %) (Table 3).

**Table 3:** Behavioral and emotional domains as a function of headache status in the children and their mothers

CBCL domains	Children No headache						Children Migraine					
	Mother No headache			Mother Migraine			Mother No headache			Mother Migraine		
	n	%	RR (95 % CI)	n	%	RR (95 % CI)	n	%	RR (95 % CI)	n	%	RR (95 % CI)
Withdrawn	19	6.4	Reference	3	6.5	1.0 (0.3–3.3)	22	8.9	1.4 (0.8–2.5)	7	3.9	0.6 (0.3–1.4)
Somatic	9	3.0	Reference	1	2.2	0.7 (0.1–5.5)	50	20.3	**6.7 (3.6–13.3)**	43	23.8	**7.8 (3.9–15.6)**
Anxiety-Depressive	10	3.4	Reference	2	4.3	1.3 (0.3–5.7)	31	12.6	**3.7 (1.9–7.4)**	33	18.2	**5.4 (2.7–10.7)**
Social	16	5.4	Reference	2	4.3	0.8 (0.2–3.4)	18	7.3	1.3 (0.7–2.6)	22	12.2	**2.2 (1.2–4.2)**
Thought	8	2.7	Reference	1	2.2	0.8 (0.1–6.3)	9	3.7	1.3 (0.5–3.5)	7	3.9	1.4 (0.5–3.9)
Attention	18	6.1	Reference	4	8.7	1.4 (0.5–4.0)	39	15.9	**2.6 (1.5–4.4)**	34	18.8	**3.1 (1.8–5.3)**
Delinquent	15	5.1	Reference	2	4.3	0.9 (0.2–3.6)	19	7.7	1.5 (0.8–2.9)	10	5.5	1.1 (0.5–2.4)
Aggressive	12	4.1	Reference	0	0.0	0.0	7	2.8	0.7 (0.3–1.8)	8	4.4	1.1 (0.4–2.6)
Internalizing	53	17.9	Reference	13	28.3	1.6 (0.9-2.7)	121	49.2	**2.7 (2.1–3.6)**	98	54.1	**3.0 (2.3–4.0)**
Externalizing	47	15.9	Reference	5	10.9	0.7 (0.3–1.6)	40	16.3	1.0 (0.7–1.5)	33	18.2	1.1 (0.8–1.7)
Total	43	14.5	Reference	7	15.2	1.0 (0.5–2.2)	80	32.5	**2.2 (1.6–3.1)**	60	33.1	**2.3 (1.6–3.2)**

	Children No headache						Children Migraine					
	Mother No headache			Mother Headaches on 15 days or more			Mother No headache			Mother Headaches on 15 days or more		
Internalizing	53	17,9	Reference	8	42,1	**2.3 (1.3–4.2)**	121	49,2	**2.7 (2.1–3.6)**	21	53,8	1.1 (0.8–1.5)
Externalizing	47	15,9	Reference	4	21,1	1.3 (0.5–3.3)	40	16,3	1.1 (0.7–3.2)	5	12,8	0.8 (0.3–1.9)
Total	43	14,5	Reference	6	31,6	**2.1 (1.1–4.4)**	80	32,5	**2.2 (1.6–3.1)**	14	35,9	1.1 (0.7–1.7)

The values in bold are statistically significant

Two contrasts are worth emphasizing from Table 3. First, children with migraine had significantly different CBCL scores relative to children without migraine and this did not vary significantly as function of headache status in the mother. However, when children had no headaches, frequency of headaches in the mother mattered. Indeed, children without headaches of mothers with frequent headaches were more likely to have abnormal scores of internalizing (42.1 vs. 17.9 %) and total score symptoms (31.6 vs. 14.5 %) (Table 3).

In multivariate analyses, headache status in the mother and headache frequency in the mother did not predict CBCL scores in children with migraine, but predicted in children without migraine (*p* < 0.01).

## Discussion

Children with early onset migraine seem to be more likely to have behavioral displays relative to children without migraine [32]. Conflicting explanations for the association exist. While some believe it reflects shared biological predisposition (e.g., brain neurotransmitter dysfunctions would predispose to migraine and these manifestations), others believed that shared environmental exposure could explain it [32]. Finally, others believe that the comorbid conditions have a direct relationship (e.g., frequency of headaches predispose to behavioral abnormalities) [33].

Three important findings emerged from our study. First, migraine influences the CBCL scores in children, which was expected and largely confirmatory by clinical [16–19] and populational studies [20, 21]. Furthermore in children with migraine, headache status or headache frequency in the mother do not influence the CBCL scores. Third and more interesting, headache frequency in the mother was associated with internalizing symptoms in children without headaches.

To the best of our knowledge, only one previous study have investigated the association of headache/migraine and behavior in children and their parents in a clinical sample of 200 children with chronic headaches [33]. The authors found a high prevalence of psychiatric disorders in children with headache and their parents, but a specific pattern in children with migraine in which a higher prevalence of psychiatric disorders in parents, co-occurrence of psychiatric comorbidity and headache familial recurrence could be observed suggesting possible mechanisms of familial co-transmission of migraine and psychiatric comorbidities.

Our findings suggest that the burden of migraine to the children is incredibly complex. First, migraine per se is associated with behavioral symptoms in this population and causality needs to be assessed in future studies. Second, we found that headache status in the mother is also of importance if the children do not have migraine (but not if they have).

Our study confirms and expands findings of another population study, where individuals with and without migraine and their partners were interviewed [34]. Of people with migraine living with a household partner, 50 % believed that because of their migraine, they were more likely to argue with their partners (50 %) and children (52 %), while majority (52–73 %) reported other adverse consequences for their relationships with their partner and children, and at work. Participating partners partly confirmed these findings: 29 % felt that arguments were more common because of headaches and 20–60 % reported other negative effects on relationships at home. Our study adds by directly measuring psychological symptoms in the children.

We raise one very important cautionary note: we have not assessed psychiatric status in the mother and headache in the father. Therefore, although we adjusted for income, demography and headache parameters, we have not adjusted for psychiatric maternal status. Accordingly, it may well be that psychiatric maternal status predicts proband psychiatric status, and since migraine and psychiatric disorders are comorbid, we are yet missing one piece to disentangle the mechanisms of comorbidity.
